# Cutaneous transcriptome analysis in NIH hairless mice

**DOI:** 10.1371/journal.pone.0182463

**Published:** 2017-08-07

**Authors:** Zhong-Hao Ji, Jian Chen, Wei Gao, Jin-Yu Zhang, Fu-Shi Quan, Jin-Ping Hu, Bao Yuan, Wen-Zhi Ren

**Affiliations:** Department of Laboratory Animals, College of Animal Sciences, Jilin University, Changchun, Jilin, China; University of Alabama at Birmingham, UNITED STATES

## Abstract

Mice with spontaneous coat mutations are ideal animal models for studying skin development and tumorigenesis. In this study, skin hair growth cycle abnormalities were examined in NIH hairless mice 42 days after birth (P42) by using hematoxylin-eosin (H&E) staining. To examine the gene expression patterns in the skin of mutant mice, the dorsal skin of P42 female NIH mice and NIH hairless mice was sequenced by RNA-Seq, and 5,068 differentially expressed genes (DEGs) were identified (false discovery rate [FDR] ≥ 2, P < 0.05). A pathway analysis showed that basal cell carcinoma, the cell cycle and the Hippo, Hedgehog and Wnt signaling pathways were up-regulated in NIH hairless mice. Previous studies have shown that these pathways are closely associated with cell proliferation, cell cycle, organ size and cancer development. In contrast, signal transduction, bacterial and parasitic infection, and receptor-mediated pathways, including calcium signaling, were down-regulated in NIH hairless mice. A gene interaction network analysis was performed to identify genes related to hair follicle development. To verify the reliability of the RNA-Seq results, we used q-PCR to analyze 12 key genes identified from the gene interaction network analysis, including eight down-regulated and four up-regulated genes, and the results confirmed the reliability of the RNA-Seq results. Finally, we constructed the differential gene expression profiles of mutant mice by RNA-Seq. NIH hairless mice exhibited abnormalities in hair development and immune-related pathways. *Pik3r1* and *Pik3r3* were identified as key genes, laying the foundation for additional in-depth studies of hairless mice.

## Introduction

Hair follicle growth and development have a certain periodicity and include three stages: anagen, catagen and telogen. The control of these processes is important in hair generation and degradation [1, 2]. Mice with spontaneous coat mutations are useful animal models for the study of hair follicle development and hair growth [3–5], and studies of skin follicle growth and development have received increasing attention [6, 7]. Currently, researchers are focusing on the growth phenotype and changes in hair growth caused by certain mutations as well as the biological principles and molecular mechanisms underlying these characteristics [8, 9]. The use of mice with spontaneous coat mutations and targeted genetic engineering will provide invaluable information for the study of the mechanisms and principles of hair development disorders [10].

In our previous study, selected biological characteristics of NIH hairless mouse were studied, thereby providing a research basis for the study of this mouse model with a mutation affecting the coat and hair.[11]. To understand the hair development state, we observed the skin structure at the time points embryonic stage 16.5(E16.5), postnatal day 3(P3), P7, P12, P16, P21, P28, P33, P37, P42, and P60 by H&E staining (although only the results of P7 and P42 are shown), and we found significant differences between the phenotypes and pathology of the skin of the mice at P42. Through inbreeding, we established a mutant mouse strain with hair follicle development and growth cycle disorders (NIH hairless mice), making this strain a good animal model for studying human diseases.

Transcriptome sequencing is used to study transcripts of particular tissues or organs of a species in a particular state and is superior to microarrays in terms of information volume and accuracy [12]. By constructing a transcriptome library, we can obtain information about transcripts and alternative splicing and can determine the differential expression patterns of specific genes [13–16].

In the present study, the expression patterns of skin-related genes in NIH hairless mice at P42 were examined by RNA-Seq, and these patterns were then compared with those in NIH normal mice. Through Gene Ontology (GO), pathway and differentially expressed gene (DEG) interaction network analyses, the potential associations between these DEGs and hair follicle development were analyzed to identify key genes important in the development of the mouse hair follicle. The differences in some DEGs were then validated by q-PCR.

## Materials and methods

### Ethics statement

All animal experiments were approved by the Institutional Animal Care and Use Committee (IACUC) of Jilin University. The animal experiments were performed in accordance with the requirements of the Experimental Animal Ethics and Welfare guidelines (Permit Number: 20160205).

### Histological analysis of NIH normal and NIH hairless mice

SPF-grade female NIH hairless and NIH normal mice of the strain SCXK-2011-0003 (201600017738) were originally purchased from the Institute of Biological Products Co., Ltd(Changchun, China). Mice were bred in the Laboratory Animal Center of Jilin University. In the place of the back midline's right and one centimeter above the tail, scissor a lump of skin whose length is around one centimeter and width is about 0.25 centimeter at postnatal day 7 (P7) and P42. Skin samples were embedded in paraffin, fixed, cut into 6-μm slices, and subjected to hematoxylin-eosin (H&E) staining following a standard method. Images were acquired on an optical microscope (Olympus, Tokyo, Japan).

### Sample preparation

Six-week-old female NIH hairless and NIH normal mice were sacrificed by cervical dislocation (n = 5 per group). In the place of the back midline's right and one centimeter above the tail, scissor a lump of skin whose length is around 0.5 centimeter and width is about 0.25 centimeter, and total RNA was extracted with TRIzol reagent following the manufacturer’s instructions (Invitrogen, Carlsbad, CA, USA). The RNA quality was determined on a Bioanalyzer 2200 (Agilent), and the samples were stored at −80°C. RNA with an RIN > 6.0 was used for cDNA library construction.

### GO analysis

GO analysis was applied to analyze the main functions of the DEGs according to Gene Ontology [17]. Fisher’s exact tests and χ^2^ tests were used to classify the GO categories, and the false discovery rate (FDR) [18] was calculated to correct the P-value (smaller FDRs indicated a smaller error in judging the P-values). The FDR was defined as FDR = 1—N_k_ / T, where N_k_ refers to the number of Fisher’s test P-values that were less than the χ^2^ test P-values. The P-values for the GO annotations of all DEGs were calculated. Enrichment provides a measure of the significance of the function: as the enrichment increases, the corresponding function is more specific, which helps us determine the GO categories with more concrete descriptions. Within the significant category, the enrichment Re was given by Re = (n_f_ / n) / (N_f_ / N), where n_f_ is the number of DEGs within the particular category, n is the total number of genes within the same category, N_f_ is the number of DEGs in the entire microarray, and N is the total number of genes in the microarray [19].

### Pathway analysis

A pathway analysis was performed to determine the significant DEG pathways. Pathway annotations were downloaded from the Kyoto Encyclopedia of Genes and Genomes (KEGG) database (http://www.genome.jp/Kegg/). Fisher’s exact test was used to identify the enriched pathways. The resulting P-values were adjusted using the Benjamini-Hochberg (BH) FDR algorithm [20]. Pathway categories with an FDR < 0.05 were selected for further analysis.

Enrichment provides a measure of the significance of the function; as the enrichment increases, the corresponding function is more specific, which helps us determine the significant pathways. The enrichment was calculated as enrichment = (n_g_ / n) / (N_g_ / n), where n_g_ is the number of DEGs within the particular pathway, n_a_ is the total number of genes within the same pathway, n_g_ is the number of DEGs with at least one pathway annotation, and n_a_ is the number of genes with at least one pathway annotation in the entire microarray.

### Gene interaction network analysis

The KEGG database was used to build a network of genes based on the relationships between the genes, proteins and compounds in the database [21–24].

### Co-expression network

For a comprehensive analysis of the expressed genes that takes into account all significantly differentially expressed transcripts, we used a well-described (unsupervised) methodology for gene correlation network analysis to cluster transcripts into groups of highly interconnected modules based on a topological overlap mapping method known as weighted gene correlation network analysis (WGCNA) [25].

Within the network analysis, degree centrality is the simplest and most important measure of the centrality of a gene within a network, indicating its relative importance. Degree centrality is defined as the number of links that one node has with another [26]. Moreover, to examine the network properties, W-cores were introduced in order to simplify the graph topology analysis. A W-core is a subnetwork in which all nodes are connected to at least W other genes in the subnetwork. A W-core of a protein-protein interaction network usually contains cohesive groups of proteins [26, 27]. The purpose of the network structure analysis is to locate core regulatory factors (genes) in one network, with core regulatory factors defined as those that connect the most adjacent genes and have the highest degrees. The core regulatory factors were determined by the differences in degree between two class samples [28]. These factors always have the largest differences in degree.

### Real-time q-PCR analysis

Real-time q-PCR was performed to validate the gene expression data obtained from the RNA-Seq analysis. Total RNA was reverse-transcribed using TRIzol (Invitrogen, Carlsbad, CA, USA) according to the manufacturer’s instructions. Approximately 1 μg of total RNA was used for first-strand cDNA synthesis. Each q-PCR contained 2 μL of DNA template, 12.5 μL of SYBR Fast q-PCR Mix (Takara, Tokyo, Japan), 8.5 μL of ddH_2_O, 1 μL of upstream primer F and 1 μL of downstream primer R in a final volume of 25 μL. The q-PCR reaction parameters included a 15-s denaturation step at 95°C and 40 cycles of 95°C for 5 s and 60°C for 30 s followed by a melting curve analysis. The primer pairs used for q-PCR amplification were designed using Integrated DNA Technologies (IDT, http://sg.idtdna.com/site). The CT value in the fluorescent quantitative reaction indirectly represents the expression level of the gene, and the relative quantification of gene expression can be achieved by comparison with a housekeeping gene. In this experiment, *Actb* was used as the internal reference, and relative gene expression was calculated using the following formula: ΔΔCt = ΔCt (case)—ΔCt (control), with the difference determined according to 2^-ΔΔCt^ [29].

### Immunohistochemistry

The proteins Pik3r1 and Pik3r3 in tissue sections from female NIH normal and NIH hairless mice at P7 and P42 were detected using the SABC method. Tissue sections (6 μm) from each group were dehydrated in xylene and a graded ethanol series, and other reagents were added in the following order: primary antibody [Pik3r1 (1:300), Pik3r3 (1:300); rabbit polyclonal antibodies, BBI, Shanghai, China)], biotinylated secondary antibody, SABC reagents and DAB solution (Wuhan Boster Co, Wuhan, China.). The sections were then rinsed in distilled water, dehydrated using alcohol and xylene, mounted on coverslips using permanent mounting medium and observed under an optical microscope equipped with a 10× objective (Olympus, Tokyo, Japan). For negative controls, the slides were treated with PBS instead of the primary antibody.

## Results

### Phenotypic descriptions

**Fig 1:**
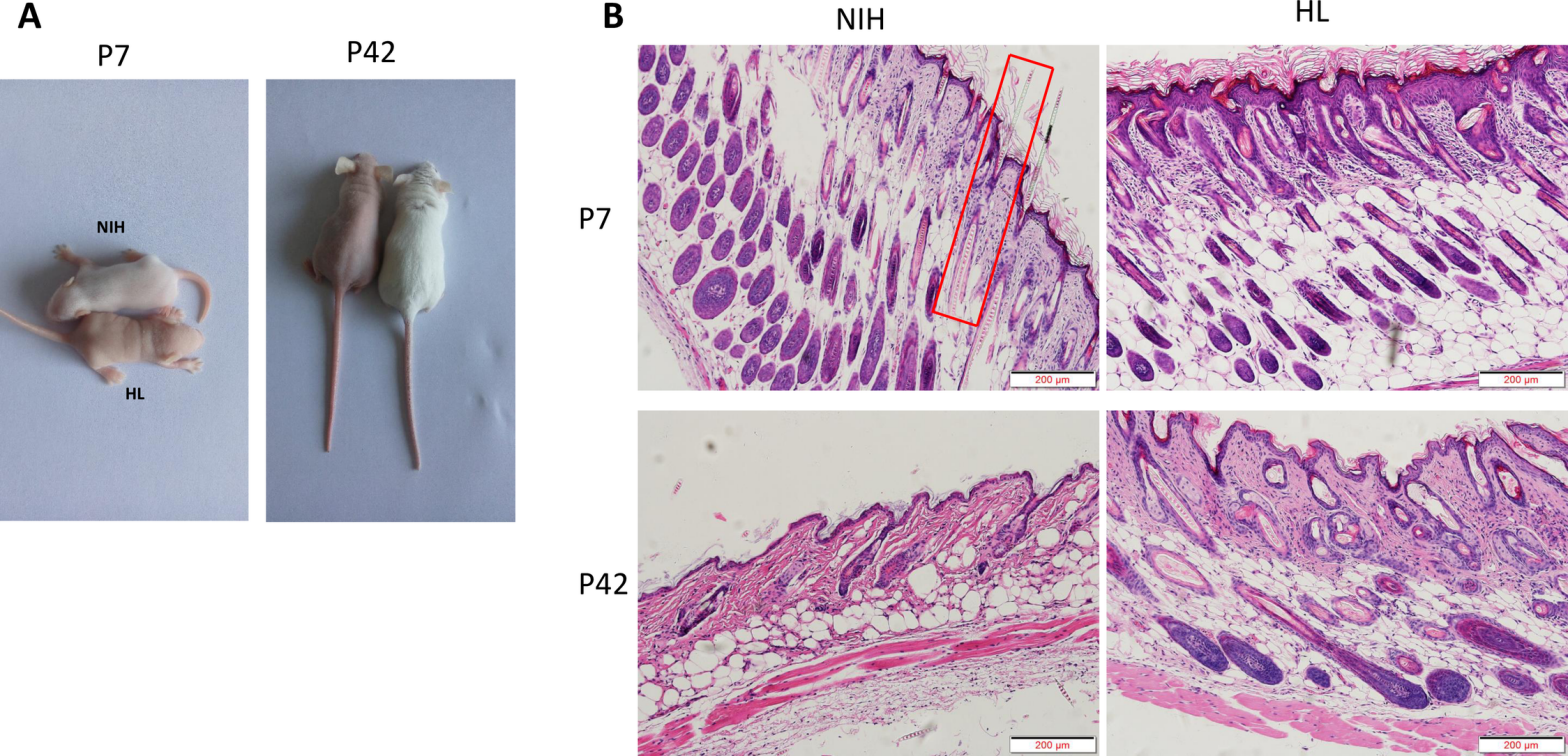
Phenotypes of NIH hairless and NIH normal mice. A: Hair loss in seven- and 42-day-old HL (NIH hairless) and NIH (NIH normal) mice. At P7, body hair completely covers NIH mice, whereas HL mice have completely bare pink skin. B: Histological features of HL mice at P7 and P42. H&E-stained sections show the arrangement of neat, normally growing hair follicles (HFs) and hair shafts in NIH mice at P7 (displayed in the red box). In HL mice, the hair shaft cannot penetrate the epidermis. At P42, the HFs of NIH normal mice exhibited normal cycling. In contrast, the subcutaneous fat layer of HL mice contains abnormal hair follicles and hair follicle development cycle abnormalities. Bar = 200 microns.

NIH normal and NIH hairless mice appeared identical at birth. At P7, NIH mice were completely covered with body hair, whereas the NIH hairless (HL) mice retained their bare pink skin (Fig 1A). With age, the HL mice grew sparse hair and had clearly distinct histological features at P7 and P42. The H&E-stained sections of the NIH mouse skin showed neatly arranged hair follicles (HFs) and normal growth at P7. At P42, the HFs of NIH normal mice exhibited normal cycling. Conversely, the subcutaneous tissue fat layer of HL mice contained abnormal hair follicles and showed abnormal hair follicle development (Fig 1B).

### RNA-Seq analysis of NIH normal and NIH hairless mice

RNA-Seq libraries were constructed from NIH normal and HL mice. Fast-QC (http://www.bioinformatics.babraham.ac.uk/projects/fastqc/) software was used to evaluate the quality of the sequencing data, which included the base quality value distribution, position distribution, GC content and PCR duplication content. The quality control results are shown in S1 Table. Map Splice software was used to compare the RNA-Seq data. The mapping statistics are shown in S1 Table.

### Identification of DEGs

We examined the expression of 22,426 genes. Differential gene expression analysis of the control (NIH normal mice) and case (NIH hairless mice) groups was performed using the DESeq algorithm.

**Fig 2:**
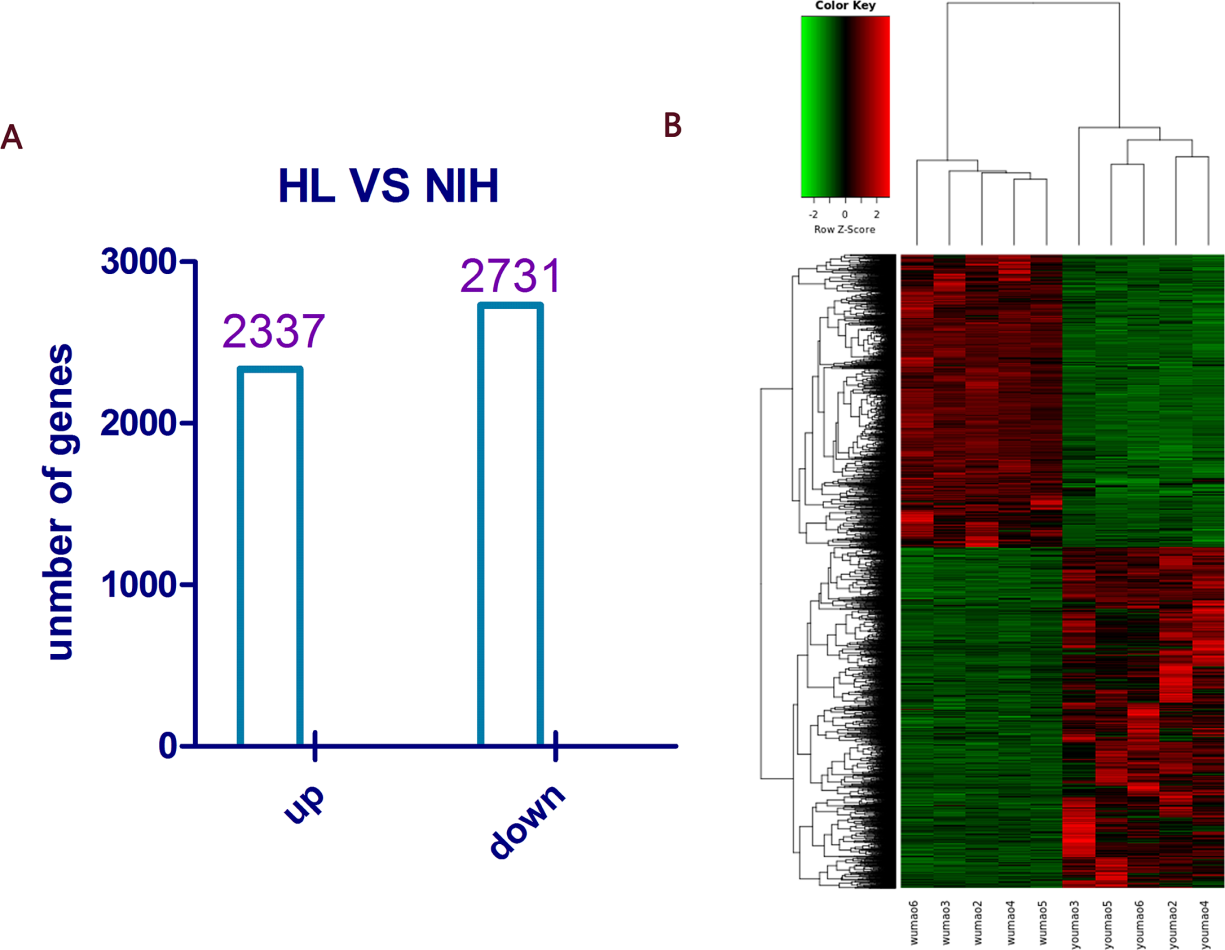
Gene expression profiles in HL mouse skin using RNA-Seq. A: In total, 5,068 DEGs (FDR ≥ 2, P < 0.05) were obtained after sequencing analysis, and these included 2,337 up-regulated genes and 2,731 down-regulated genes. B: Hierarchical clustering represents the differential expression of genes in the skin between NIH hairless and NIH normal mice. There were five repetitions within each group.

We obtained a total of 5,068 DEGs, of which 2,337 were up-regulated and 2,731 were down-regulated (Fig 2; see S1 File for details).

### GO and pathway analysis

**Fig 3:**
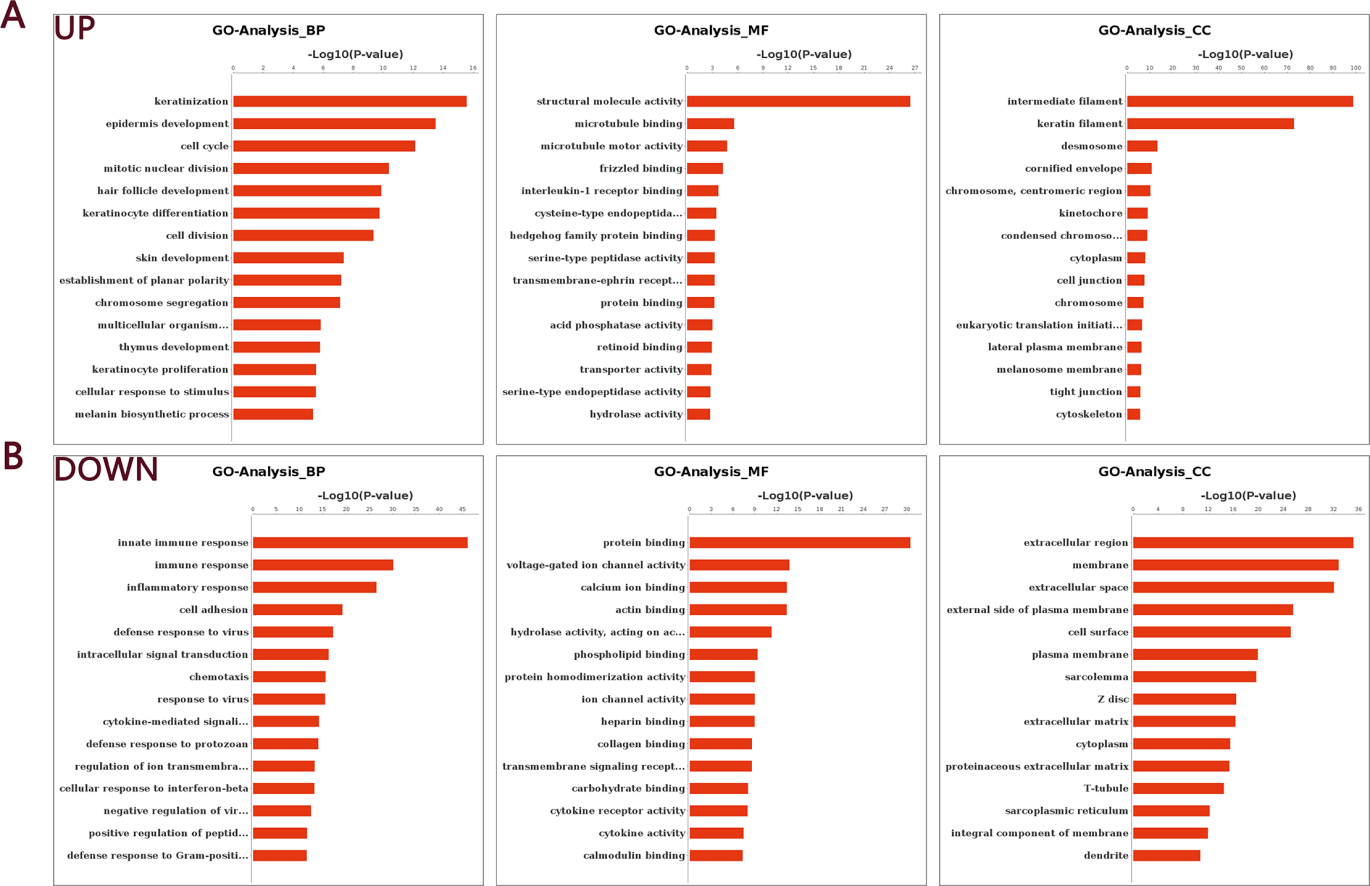
GO analysis of all differentially expressed unigenes. A: Up-regulated genes in the skin of NIH HL and NIH normal mice. B: Down-regulated genes in the skin of NIH HL and NIH normal mice.

GO enrichment analysis was performed on the RNA-Seq data. Among the 5,068 DEGs, 4,188 were annotated to 1,256 significant biological process GO categories (P < 0.05); 4,271 were annotated to 170 significant cell component GO categories (P < 0.05); and 4,152 were annotated to 334 significant molecular function GO categories (P < 0.05). As shown in Fig 3, keratinization, epidermis development, cell cycle, mitotic nuclear division, hair follicle development, keratinocyte differentiation and skin development were associated with genes that were highly expressed in NIH HL mice, whereas genes that were weakly expressed in NIH HL mice were mainly associated with the innate immune response, the immune response, the inflammatory response, cell adhesion and defense responses to virus infection (see S2–S4 Files for details).

**Fig 4:**
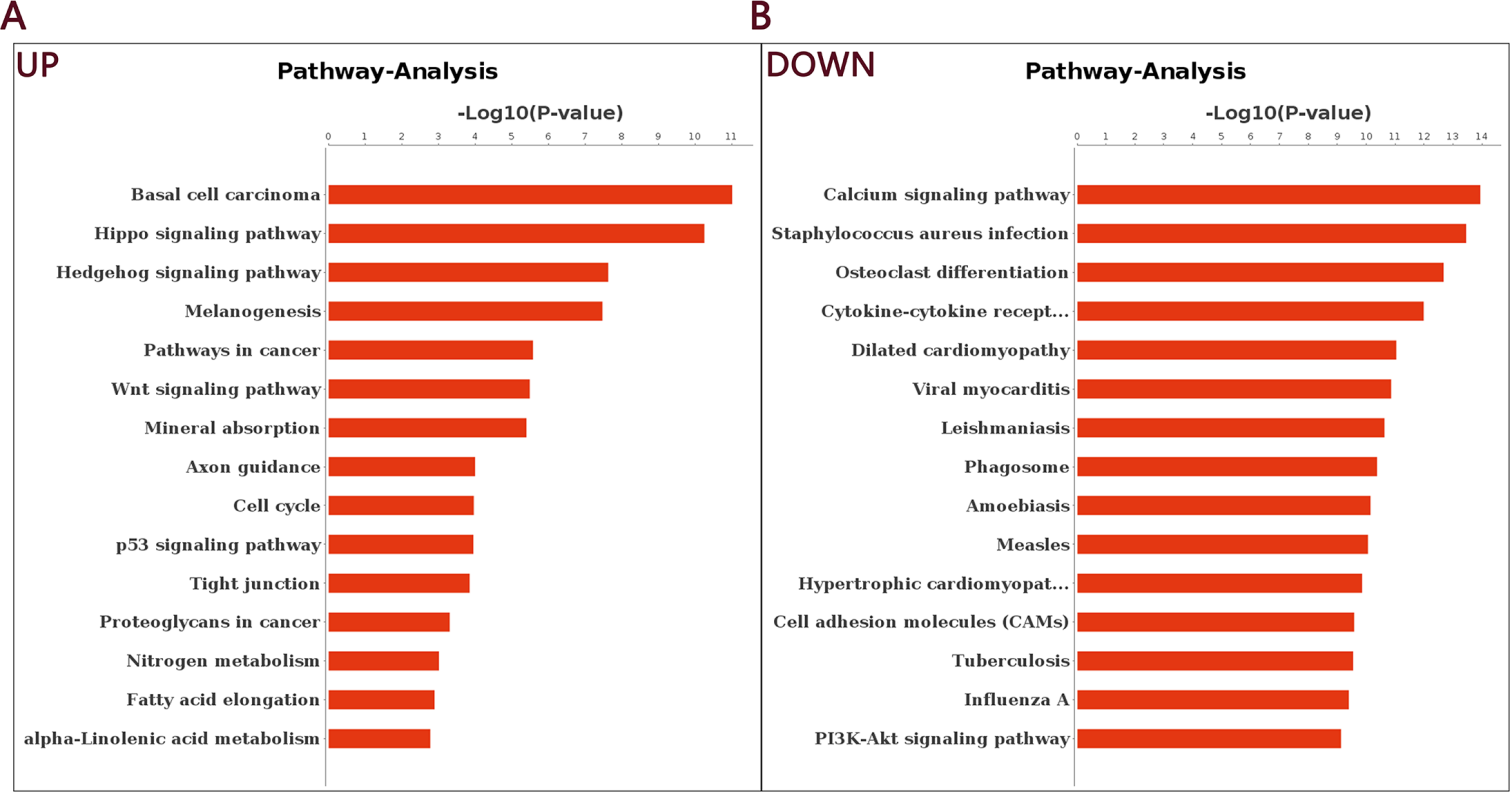
Pathway analysis of all differentially expressed unigenes. A: Up-regulated genes in the skin of NIH HL and NIH normal mice. B: Down-regulated genes in the skin of NIH HL and NIH normal mice.

We then used the KEGG database to analyze pathways associated with the DEGs and identified 37 up-regulated pathways and 118 down-regulated pathways. The comparison between NIH hairless and NIH normal mice showed that the DEGs were associated with immunity, cell cycle, apoptosis, cancer and microbial infection. Fig 4 presents the up- and down-regulated pathways obtained from pathway analysis and sorted by confidence level. The GO and pathway analyses allowed us to preliminarily classify the DEGs, which will provide important information for the screening of functional genes (see S5 File for details).

### Weighted gene co-expression network analysis

**Fig 5:**
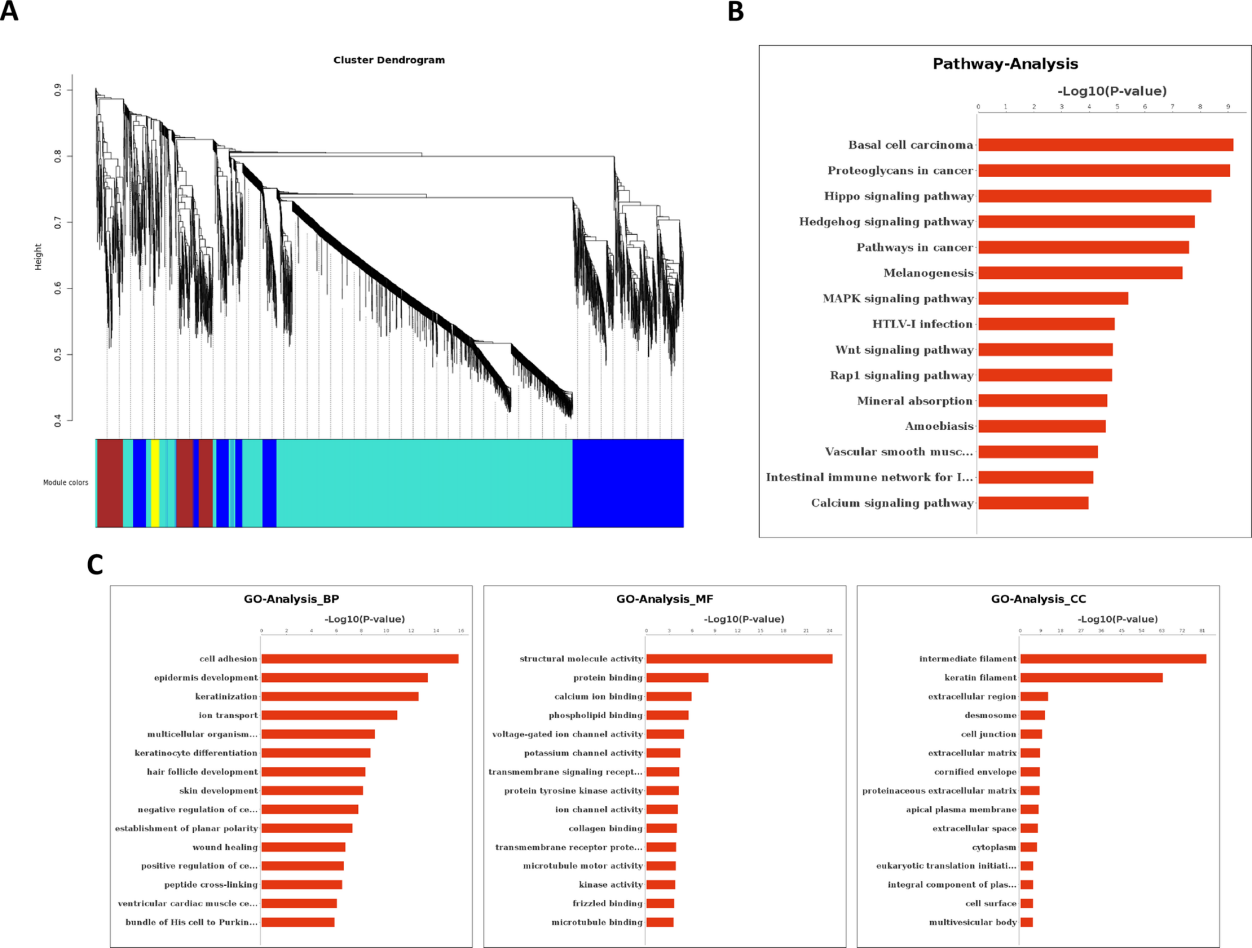
Results of the weighted co-expression analysis of DEGs using the WGCNA algorithm. A: Network heat map. The branch in the hierarchical cluster tree corresponds to the module. B: Relationships between HL and NIH module traits. Different colors represent different classification modules. C: GO analysis of DEGs in the turquoise module. D: Pathway analysis of DEGs in the turquoise module.

The WGCNA clustered 5,068 transcripts into four co-expression modules. The module conservation across all datasets is shown as a dendrogram in (Fig 5). Based on WGCNA convection, the four enriched modules were colored turquoise, yellow, brown and blue. Among them, the turquoise module (Fig 5) represents the genes of interest and shows the 838 DEGs identified and the results of the GO and pathway analyses (see S6 File for details).

### Gene interaction network and pathway interaction network analyses

**Fig 6:**
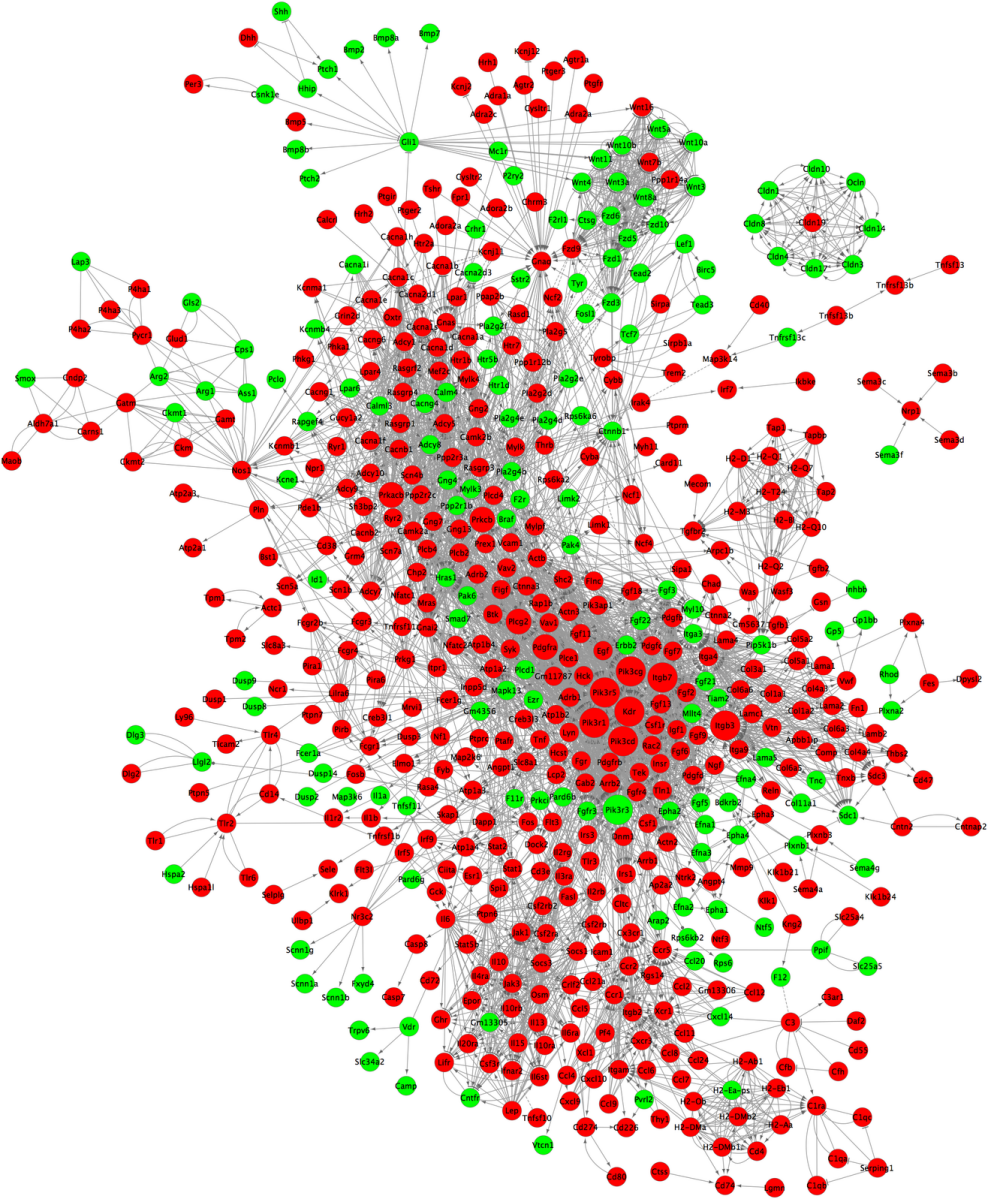
Gene interaction network and pathway interaction network analyses. For genes that are not associated with human diseases, larger dots correspond to higher degrees of participation in the interaction network. Red represents down-regulated genes, and green represents up-regulated genes.

**Fig 7:**
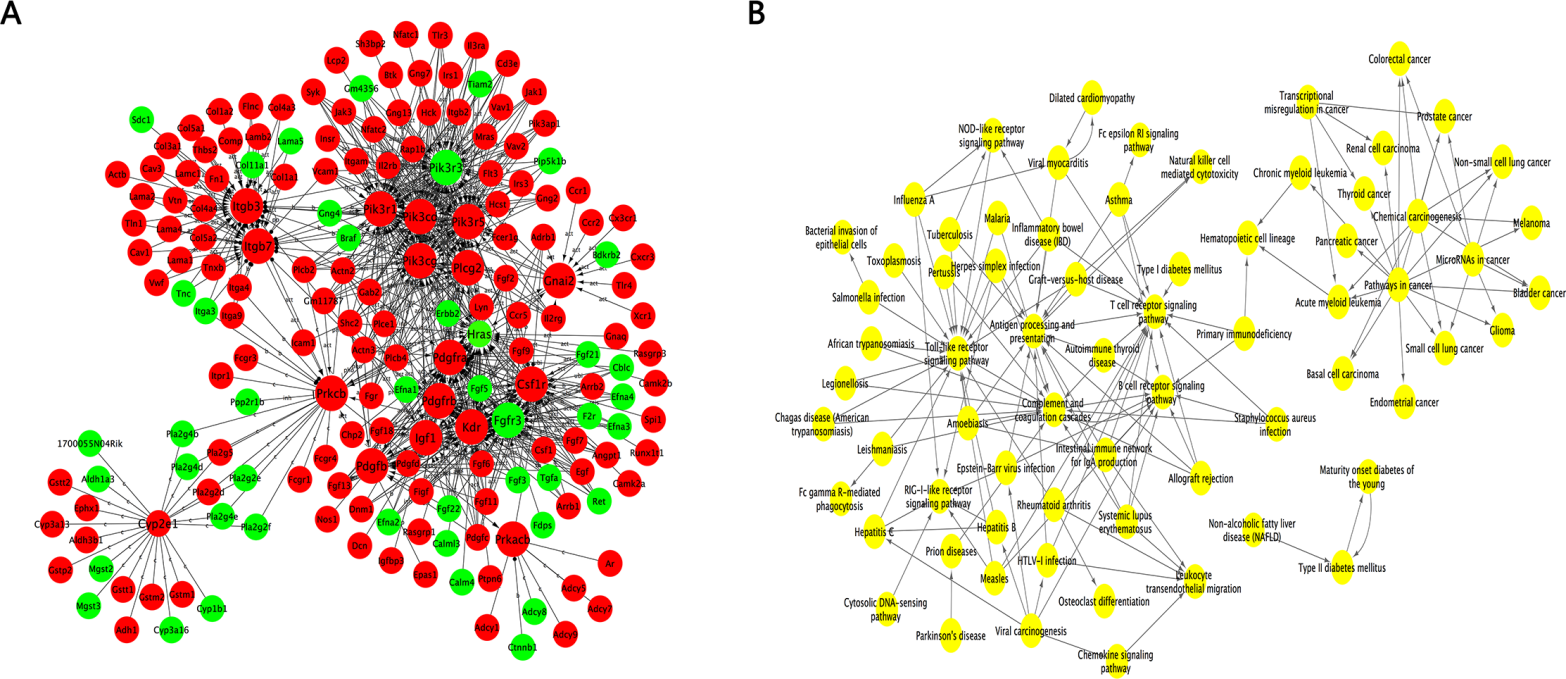
Gene interaction network and pathway interaction network analyses. A: For genes that are associated with human diseases and immune pathways, larger dots correspond to higher degrees of participation in the interaction network. Red represents down-regulated genes, and green represents up-regulated genes. B: The pathway interaction network between human diseases and organismal systems. Circular nodes represent pathways, and an arrow between two nodes represents an interaction target between pathways.

To further study the interactions between genes and the internal links between pathways, we built two gene interaction networks and one pathway interaction network based on the KEGG database. We first chose genes that were not associated with human diseases to build the gene interaction network (Fig 6). In total, 628 DEGs were included in this network, in which red represents the down-regulated genes and green represents the up-regulated genes in the experimental group. The degree is arranged based on the degree of involvement of the genes in the network. Genes in the PI3K family, including *Pik3r1*, *Pik3r3* and *Pik3cd*, were identified as core genes. We also analyzed the differences in DEGs associated with human diseases. The gene interaction network (Fig 7A) showed that the PI3K family members were still situated at the core of the network. We also built a pathway interaction network between human diseases and organismal systems (Fig 7B).

### Confirmation of RNA-Seq results by q-PCR

**Fig 8:**
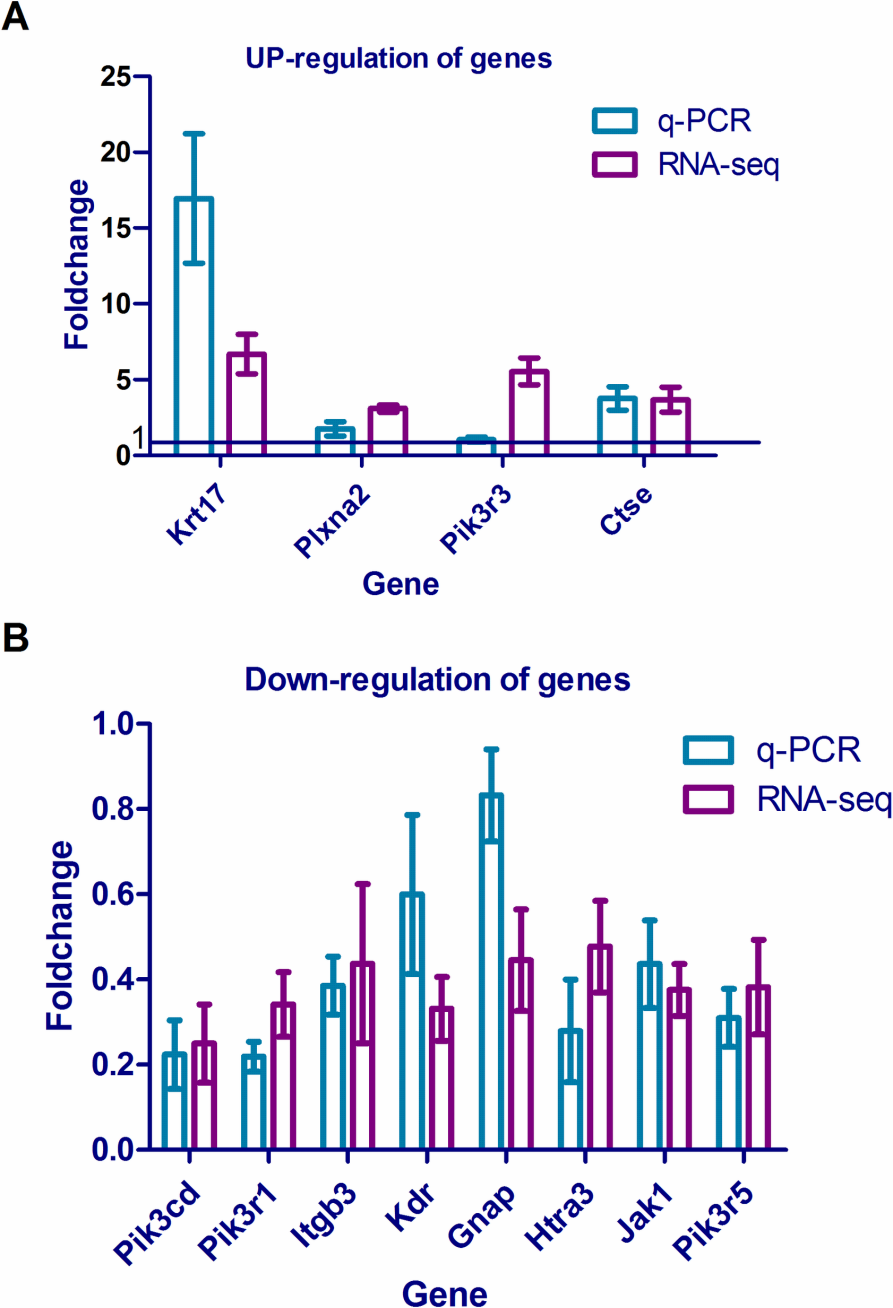
Confirmation of RNA-Seq results by q-PCR. A: Four up-regulated genes (*Krt17*, *Plxna2*, *Pik3r3* and *Ctse*). B: Eight down-regulated genes (*Pik3r1*, *Pik3cd*, *Itgb3*, *Kdr*, *Gnaq*, *Htra3*, *Jak1* and *Pik3r5*).

To ensure that our sequencing data were true and reliable, we selected several relatively important genes obtained from the gene interaction network analysis, four up-regulated genes (*Krt17*, *Plxna2*, *Pik3r3* and *Ctse*) and eight down-regulated genes (*Pik3r1*, *Pik3cd*, *Itgb3*, *Kdr*, *Gnaq*, *Htra3*, *Jak1* and *Pik3r5*) (12 total), which were verified by q-PCR (Fig 8). The results obtained from the two methods were consistent for each gene (see S7 File for details).

### Expression of Pik3r1 and Pik3r3 proteins in tissue sections

**Fig 9:**
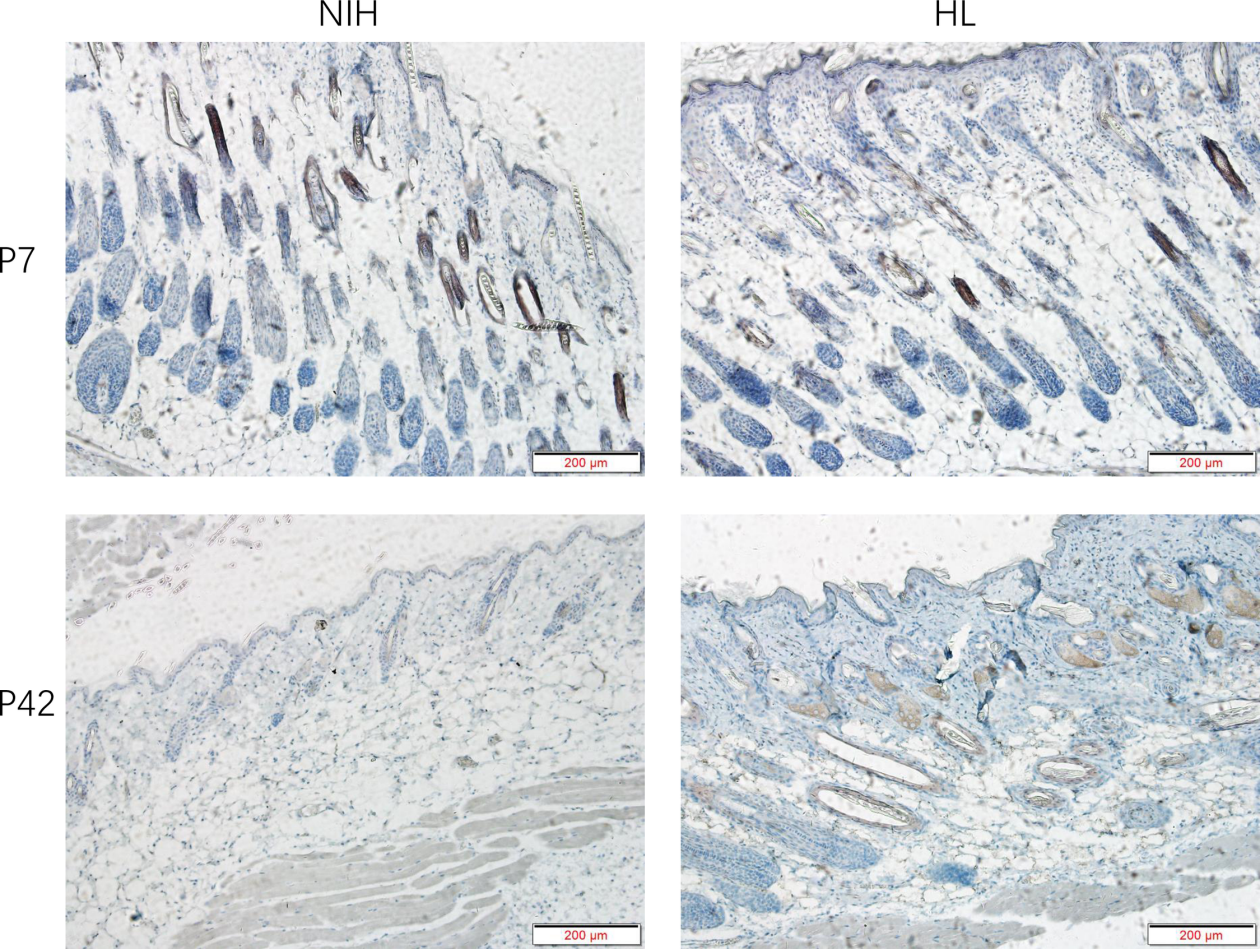
Expression of Pik3r1 protein in tissue sections. The Pik3r1 antibody shows signals in partial hair follicles. In addition, the sebaceous glands also show some Pik3r1 expression.

**Fig 10:**
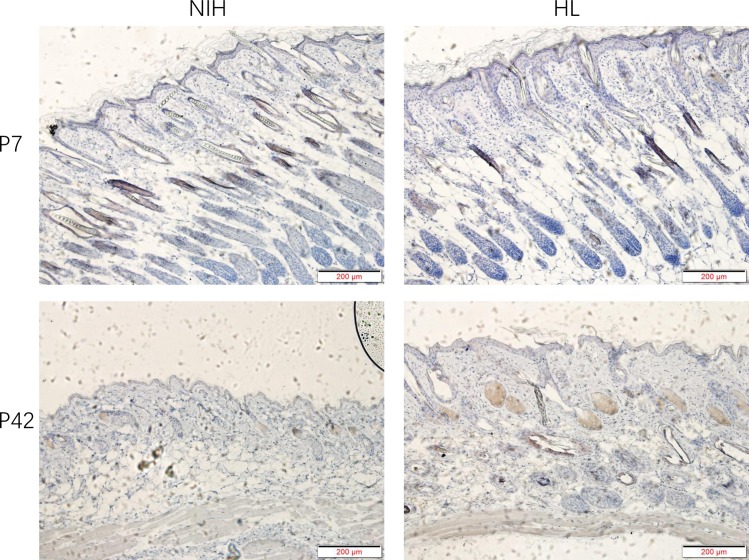
Expression of Pik3r3 protein in tissue sections. The Pik3r3 antibody shows an expression pattern similar to that of the Pik3r1 antibody.

To study the relationship between the *Pik3r1* and *Pik3r3* genes and hair development, we used immunohistochemistry to observe the expression of these two proteins in the skin. We found that the Pik3r1 and Pik3r3 proteins were mainly expressed in the hair follicles, even though the sebaceous glands also showed some levels of Pik3r1 and Pik3r3 expression. Interestingly, although these two proteins were both found in some hair follicles and were not expressed in others, we hypothesized that this result was related to whether the hair follicles were active or inactive. However, the specific evidence for this phenomenon requires further in-depth studies (Figs 9 and 10).

## Discussion

Mice with spontaneous coat mutations are useful animal models for the study of hair follicle development and hair growth [30]. The skin is the body’s first immune barrier. The functions of the skin are integrated into the skin immune, pigmentary, epidermal and adnexal systems and are in continuous communication with the systemic immune, neural and endocrine systems [31]. Mice with skin mutations often exhibit immune dysfunctions, which indicates that bodily infections also have important implications for immunity [32]. Previous studies have shown that the skin is affected by the regulation of corticotropin-releasing factor (CRF), which produces some direct or indirect phenotypic effects that can regulate epidermal barrier function and the skin immune, pigmentary, adnexal, and dermal functions necessary to maintain local and systemic homeostasis [33, 34]. To date, several hairless mouse strains have been used to investigate immune responses, tumorigenesis, skin development and other research questions. Some such models are nude mice with *Foxn1* gene mutations; these mice lack thymic cell immunity and are used for tumor modeling and for evaluating drug effects [35–37]. Another model is the hairpoor mouse (hr^hp^), which was generated via N-ethyl-N-nitrosourea (ENU) mutagenesis and has a coat of abnormal hairs [55]; this mouse was found to have a point mutation in the hairless gene, making it a good animal model for studying the human familial genetic disease Marie Unna hereditary hypotrichosis (MUHH). We found that NIH hairless mice exhibit hair follicle developmental disorders in the first hair growth cycle. Additionally, other researchers have reported progressive hair loss and abnormal hair growth cycles in these mice.

We used RNA-Seq high-throughput sequencing technology [38–40] to examine and compare the phenotypes and skin structures of six-week-old female NIH normal and NIH HL mice. We found that keratinization, epidermis development, hair follicle development and skin development were up-regulated in NIH hairless mice. H&E staining of P42 skin slices showed that in NIH normal mice, the second hair growth cycle was in telogen, whereas in HL mice, the hair growth cycle was still in anagen. The innate immune response, immune response, viral response and other pathways closely associated with immune defense showed downward trends, indicating abnormal immune function in NIH hairless mice [41–43]. These results are consistent with the spontaneous tumor formation that occurs when breeding NIH hairless mice. Pathway analysis showed that genes involved in basal cell carcinoma, the cell cycle and the Hippo, Hedgehog and Wnt signaling pathways were up-regulated in HL mice; these pathways are associated with cell proliferation, growth, cycle, organ size and cancer [30, 44]. In contrast, genes involved in signal transduction, receptor-mediated signaling (e.g., calcium, cytokine-cytokine receptor, and PI3K-Akt signaling) and bacterial and parasitic infection (e.g., *Staphylococcus aureus* infection) were down-regulated in HL mice.

Because many genes might affect the screening of core genes and the construction of the interaction network, we used WGCNA to classify genes based on their expression patterns and to categorize the modules by color (the turquoise modules are closely related to our research aims). To further understand the importance of gene and pathway interactions and to screen for key genes and pathways that play significant roles in the case group (NIH hairless mice), we built two gene interaction networks and one pathway interaction network based on the direct or systemic interactions assigned between pathways in the KEGG database. Based on the degrees of involvement of the genes in the network, *Pik3r1*, *Pik3r3*, *Pik3cd*, *Kdr*, *Itgb3* and *Itgb7* were identified as key genes.

Previous studies have shown that the PI3K / AKT signaling pathway is involved in cell regulation processes, including cell growth, proliferation, transformation, epithelial-to-mesenchymal transition and survival, and cell pathology [45–47]. Disruption of the PI3K / AKT signaling pathway can lead to cell growth, proliferation and survival disorders, thus affecting normal bodily activities. Studies of the *Pik3r1* gene have described its association with the occurrence and metastasis of cancer [48–50]. Notably, the analysis of atopic dermatitis sequencing data revealed a crucial role for *Pik3r1* in atopic dermatitis [30, 51]. However, research on its involvement in hair follicle development is lacking. Finally, we found that *Pik3r1* and *Pik3r3* proteins were specifically expressed by hair follicles.

In summary, we constructed gene expression profiles for the skin of six-week-old NIH hairless and NIH normal mice. A bioinformatics analysis of the 5,068 DEGs revealed differences in the cell cycle, immune response and skin development. Screening of the *Pik3r1* and *Pik3r3* genes will be of great importance in our future research.

## Supporting information

S1 Table Quality control. (XLSX)

S2 Table Mapping statistics. (XLSX)

S1 File Differential gene expression analysis (FC ≥ 2, FDR < 0.05). (XLSX)

S2 File GO analysis of the differentially expressed genes (BP). (XLSX)

S3 File GO analysis of the differentially expressed genes (CC). (XLSX)

S4 File GO analysis of the differentially expressed genes (MF). (XLSX)

S5 File Pathway analysis of the differentially expressed genes. (XLSX)

S6 File Weighted gene co-expression network analysis. (XLS)

S7 File q-PCR verification. (DOCX)

S8 File Raw data for Fig 7. (XLSX)
