# Supplementary material for: Cutaneous transcriptome analysis in NIH hairless mice
Source: PLoS One. 2017 Aug 7;12(8):e0182463. doi: 10.1371/journal.pone.0182463 (PMC5546695; doi:10.1371/journal.pone.0182463)
Supplement: S7 File — (DOCX) [file pone.0182463.s009.docx]

Verification of the expression of 12 differential genes by qRT-PCR. Including 4 up-regulated genes and 8 down-regulated genes .

| Gene symbol | Expression in NIH hairless mice | Sequence of primers used in q-PCR | | NCBI Enter accession | Expression of fold change | |
| --- | --- | --- | --- | --- | --- | --- |
|  |  |  |  |  | RNA-seq | q-PCR |
| Pik3cd | down | Forward | GTGGTTGTTGACTTCTTGCTG | NM_001029837 | 0.24 | 0.22 |
|  |  | Reverse | GGAAGAGTGGCTCATACTGTG |  |  |  |
| Pik3r1 | down | Forward | ACGGTTTGGACTATGGAAGAC | NM_001024955 | 0.34 | 0.21 |
|  |  | Reverse | GTTCATGCTGTTGTTGGCTAC |  |  |  |
| Itgb3 | down | Forward | TCGTCAGCCTTTACCAGAATT | NM_016780 | 0.43 | 0.38 |
|  |  | Reverse | CGTACTTCCAGCTCCACTTTAG |  |  |  |
| Kdr | down | Forward | ATAGAAGGTGCCCAGGAAAAG | NM_010612 | 0.33 | 0.59 |
|  |  | Reverse | TCTTCAGTTCCCCTTCATTGG |  |  |  |
| Gnaq | down | Forward | GTAGATGCAATAAAGAGCTTGTGG | NM_008139 | 0.44 | 0.83 |
|  |  | Reverse | GTTGTGTAGGCAGATAGGAAGG |  |  |  |
| Htra3 | down | Forward | TGCAGAACACCGTGACAAC | NM_001042615.2 | 0.47 | 0.27 |
|  |  | Reverse | TCCTGAGTTCCCGTAATTGATG |  |  |  |
| Jak1 | down | Forward | CACTGGACAACCGAATAAATGC | NM_146145 | 0.37 | 0.43 |
|  |  | Reverse | CCAACAGATAGAAAGTCACCTCC |  |  |  |
| Pik3r5 | down | Forward | CTTCCCTGGCATCTTAGACAC | NM_177320 | 0.26 | 0.30 |
|  |  | Reverse | TGTAGGATGTCAAAGCTGTCC |  |  |  |
| Plxna2 | up | Forward | TGGGAAAGGATGTGCAGTG | NM_008882 | 3.10 | 1.76 |
|  |  | Reverse | CAGAGGCTGGTTGATGTCC |  |  |  |
| Krt17 | up | Forward | CAGCCAGAGACTACAGCG | NM_010663 | 6.68 | 16.94 |
|  |  | Reverse | CTTGGTACGGAAGTCATCGG |  |  |  |
| Pik3r3 | up | Forward | ATGCCCTATTCGACAGAACTG | NM_181585 | 5.53 | 1.05 |
|  |  | Reverse | CCTTCATTCCATTTGTGACTGC |  |  |  |
| Ctse | up | Forward | CACGGAGGTAGGGAATCATTTC | NM_007799 | 3.68 | 3.76 |
|  |  | Reverse | TGGCTCCTTGACACTTTCTC |  |  |  |
| Actb(beta-actin) |  | Forward | GTGGGAATGGGTCAGAAGG | NM_007393.4 |  |  |
|  |  | Reverse | AGCTCATTGTAGAAGGTGTGG |  |  |  |
